# Biotinylated *N*-Acetyllactosamine- and *N*,*N*-Diacetyllactosamine-Based Oligosaccharides as Novel Ligands for Human Galectin-3 †

**DOI:** 10.3390/bioengineering4020031

**Published:** 2017-04-05

**Authors:** Sophia Böcker, Lothar Elling

**Affiliations:** Laboratory for Biomaterials, Institute for Biotechnology and Helmholtz-Institute for Biomedical Engineering, RWTH Aachen University, Pauwelsstrasse 20, 52074 Aachen, Germany; s.boecker@biotec.rwth-aachen.de

**Keywords:** biotin, chemo-enzymatic synthesis, galectin-3, LacdiNAc, neo-glycoprotein

## Abstract

Galectin inhibitor design is an emerging research field due to the involvement of galectins in cancer. Galectin-3, in particular, plays an important role in tumor progression. To generate inhibitors, modifications of the glycan structure can be introduced. Conjugation of hydrophobic compounds to saccharides has proven to be promising as increased binding of galectin-3 can be observed. In the present study, we report on neo-glycans carrying hydrophobic biotin as novel ligands for human galectin-3. We modified *N*-acetyllactosamine- and *N*,*N*-diacetyllactosamine-based tetrasaccharides at the C6-position of the terminal saccharide unit using selective enzymatic oxidation and subsequent chemical conjugation of biotinamidohexanoic acid hydrazide. These neo-glycans were much better bound by galectin-3 than the unmodified counterparts. High selectivity for galectin-3 over galectin-1 was also proven. We generated multivalent neo-glycoproteins by conjugation of neo-glycans to bovine serum albumin showing high affinity for galectin-3. Compared to non-biotinylated neo-glycoproteins, we achieved high binding levels of galectin-3 with a lesser amount of conjugated neo-glycans. Multivalent ligand presentation of neo-glycoproteins significantly increased the inhibitory potency towards galectin-3 binding to asialofetuin when compared to free monovalent glycans. Our findings show the positive impact of 6-biotinylation of tetrasaccharides on galectin-3 binding, which broadens the recent design approaches for producing high-affinity ligands.

## 1. Introduction

Cell-surface-bound glycans mediate the interactions and communication between cells and their microenvironment [1,2,3,4]. Specialized proteins called lectins mediate the recognition and decoding of the glycan code presented by the cells [5]. Binding affinity of lectins toward their glycan ligand is rather low. It is dramatically increased by multivalent presentation of glycans in close proximity to each other, like nature does on glycolipids or glycoproteins [6]. These scaffolds bear multiple glycosylation sites or branched glycans. Moreover, arrangement in lipid rafts increases the multivalency, as well [7].

Lectins play crucial roles in disease-associated processes, such as virus or toxin binding, mediation of tumor angiogenesis, tumor progression, cell cycle arrest or metastasis [8,9,10]. The most ancient group of lectins are the β-galactoside binding galectins, formerly described as S-type lectins, as it was assumed that sulfhydryl groups are crucial for binding activity [11]. So far, at least 15 different human galectins are known. The only member of the chimera-type galectin family is galectins-3 (Gal-3), which acts cell-type specificly pro- or anti-apoptotic [12,13]. Moreover, it is reported that Gal-3 is strongly upregulated in many tumor cells [14,15,16]. The role of Gal-3 has been investigated extensively and found to be crucial in tumor progression, metastasis and angiogenesis [17,18,19,20]. This makes Gal-3 a potential target for anti-cancer therapy and cancer diagnosis. [21,22,23]. Recent reports suggest multiple glycan epitopes and low molecular weight molecules as potential inhibitor molecules [24,25]. The structural features of Gal-3 are an N-terminal non-lectin domain and a highly conserved C-terminal carbohydrate recognition domain (CRD). It is reported that in the presence of the natural ligand environment, Gal-3 forms a pentamer or even higher oligomers upon binding [26,27,28]. Therefore, inhibitors with multivalent presentation are prone to show the highest potency. However, a crucial point for the usage of glycan-based inhibitors is selectivity, as all galectins may recognize β-galactoside containing structures. Recently, we confirmed that the *N*,*N*-diacetyllactosamine (LacdiNAc, GalNAcβ1, 4GlcNAc) epitope acts as a selective ligand for Gal-3 compared to galectin-1 (Gal-1) [29]. This was surprising, as LacdiNAc has a rather low abundancy in mammalian cells [30,31,32] and is overexpressed in parasites [33,34]. Nevertheless, cancer type-dependent alteration of LacdiNAc expression was identified [35,36]. To enable a multivalent presentation of various glycan ligands in a natural way, we developed controlled glycosylation of bovine serum albumin (BSA) to yield neo-glycoproteins with tunable multivalency [37]. These scaffolds showed very high affinity towards galectins and could confirm the selectivity of LacdiNAc towards Gal-3. Finally, the search for novel glycan-based inhibitors of galectins led to the synthesis of neo-glycoconjugates based on *N*-acetyllactosamine (LacNAc) [38] and biotin at the C6-position of terminal galactose. Various approaches to design galectin inhibitors suggest the incorporation of hydrophobic residues into natural ligands [24,25,39,40]. However, these syntheses suffer often from complicated chemical routes, which are time consuming and give rather low yields. Our reported biotinylated neo-glycoconjugates combine a high affinity leading structure with a hydrophobic modification at the non-reducing end, synthesized by a chemo-enzymatic reaction [38].

We here report on the evaluation of 6-biotinylated LacNAc- and LacdiNAc-terminated tetrasaccharides as Gal-3 inhibitors. The carrier tetrasaccharides were synthesized by a cascade reaction with three recombinant glycosyltransferases and modified at the C6-position via enzymatic oxidation and reductive amination. Subsequently, the neo-glycans were conjugated to BSA via squaric acid diethyl ester coupling with various ligand densities. The binding affinity of Gal-3 towards immobilized and free neo-glycoproteins was investigated by solid phase ELISA-type assays and surface plasmon resonance spectroscopy (SPR). We found that Gal-3 binds with higher affinity to immobilized biotinylated glycans. We conclude that multimerization of Gal-3 may take place in a multivalent ligand environment and may be amplified by biotinylation of the glycan. Our study gives new insights into the binding of Gal-3 on multivalent scaffolds and presents an alternative approach for designing potent inhibitors.

## 2. Materials and Methods

### 2.1. Synthesis of Biotinylated Glycans (**6** and **7**)

The chemo-enzymatic synthesis of the tetrasaccharides LacNAc-LacNAc-linker-NH_2_-*t*Boc (**1**) and LacdiNAc-LacNAc-linker-NH_2_-*t*Boc (**2**) was performed with recombinant glycosyltransferases as described previously [37]. Both tetrasaccharides were biotinylated at C6-position of the terminal galactose and GalNAc, respectively, after oxidation using galactose oxidase. The overall procedure was followed as previously described [38] with some synthesis optimizations. For oxidation of 15 µmol oligosaccharide, 48 U galactose oxidase (Worthington, Lakewood, USA) and 480 U peroxidase (Merck, Darmstadt, Germany) were applied in 25 mM MES-NaOH (pH 6.0) saturated with oxygen and containing 5 mM CuCl_2_. The reaction took place over night under 1 bar oxygen pressure at room temperature. The reaction was stopped by ultrafiltration (10-kDa cut off, VivaSpin^®^500, Sartorius, Göttingen, Germany), as it is known that α,β-unsaturated aldehydes are formed by heat [38]. Analysis of oxidation reactions was performed by HPLC (LiChrospher 100 RP 18-5μ, 15% (*v*/*v*) acetonitrile, 0.1% (*v*/*v*) formic acid). The 6-aldehydes **3** and **4** were biotinylated with biotinamidohexanoic acid hydrazide (BACH, **5**, Sigma-Aldrich, Taufkirchen, Germany). Compounds **3** and **4** were incubated with 3.5 eq. of **5** for two days followed by the addition of 10–15 eq. of NaBH_3_CN (Sigma-Aldrich). The reaction mixture was stirred until no further reduction of biotinylated oligosaccharides **6** and **7** occurred. The progress was monitored via HPLC (Multokrom 100-5µ C18 250 mm × 4 mm, gradient separation using 11–50% (*v*/*v*) acetonitrile over a time course of 50 min at a flow rate of 0.5 mL/min). **6** and **7** were purified by preparative HPLC (Eurospher 100-10µ C18) using the same method and analyzed by LC-ESI-MS.

### 2.2. Synthesis of Compounds **11** and **12**

Deprotection under acidic conditions (1 M HCl) of **6** and **7** yielded **8** and **9**. The present amino group was used to couple squaric acid diethyl ester (**10**, Sigma-Aldrich) to the tetrasaccharides as described previously [37] with optimized conditions for this application. Glycans **8** and **9**, respectively, were incubated with 4-fold molar excess of **10** and triethylamine in 50% aqueous ethanol including 20 mM HEPES (4-(2-hydroxyethyl)-1-piperazineethanesulfonic acid) (pH 8.0) for one hour at 4 °C and 450 rpm. The products **11** and **12** were directly purified by preparative HPLC using the gradient separation method described above. Mass analysis of the products was done by LC-ESI-MS and concentration determination by HPLC and absorbance measurement at 254 nm with calibration for GlcNAc conjugated to **10**.

### 2.3. Conjugation of Glycans to Bovine Serum Albumin

Active charcoal delipidated BSA was used as the base for the synthesis of neo-glycoproteins as previously described [37]. Briefly, BSA (Carl Roth, Karlsruhe, Germany) and an appropriate amount of **11** or **12** were mixed in sodium tetraborate buffer (40 mM, pH 9.0) and incubated at room temperature and 450 rpm for up to ten days. To reduce the consumption of **11** and **12**, fed-batch mode was chosen beginning with a 1.5 molar excess compared to BSA. With desired higher modification degrees, increased additions of **11** and **12**, respectively, were done after different time points. Purification of the products **13a**–**f** and **14a**–**f** was performed by ultrafiltration, and protein concentrations were determined by the Bradford assay (Roti^®^-Quant, Carl Roth, Karlsruhe, Germany) calibrated with BSA.

### 2.4. SDS-PAGE and Streptavidin Blot

The molecular mass of the neo-glycoproteins was analyzed by SDS-PAGE followed by streptavidin blot. Here, protein amounts of 1 µg for Coomassie staining and 0.5 µg for blotting were applied using 8% gels and constant current of 25 mA. The proteins transferred to the PVDF-membrane were detected by incubation with peroxidase-conjugated streptavidin (Roche, Mannheim, Germany).

### 2.5. Expression and Purification of Recombinant Galectins

Human His_6_Gal-1C2S and human His_6_Gal-3 were expressed as described previously [37,41,42]. For the expression of the proteins, *Escherichia coli* Rosetta (DE3) pLysS (Novagen/Merck, Darmstadt, Germany) was used.

Disruption of cultivated cells and following galectin purification by immobilized metal affinity chromatography (IMAC, Ni^2+^-NTA) was performed as described elsewhere [37]. Galectins were stored in phosphate-buffered saline containing 2 mM EDTA (EPBS, pH 7.5) at 4 °C, His_6_Gal-1C2S in the presence of 10% (*v*/*v*) glycerol. Protein concentrations were measured by the Bradford assay using BSA for calibration.

### 2.6. Galectin Binding Assay with Immobilized Glycans and Neo-Glycoproteins

The synthesized glycans and neo-glycoproteins were analyzed for binding of Gal-3 and Gal-1 in 96-well microtiter plate formats [37,41]. Glycans **11** and **12**, as well as the corresponding LacNAc-LacNAc-linker-NH_2_ **1a** and LacdiNAc-LacNAc-linker-NH_2_ **2a** (deprotected forms of **1** and **2**) were immobilized via the amino group in aminoreactive microtiter plates (Immobilizer Amino, Nunc, Wiesbaden, Germany). The immobilization of 5 nmol glycan in sodium carbonate buffer (100 mM, pH 9.6) was done overnight. For immobilizing neo-glycoproteins, 5 pmol protein were incubated in PBS (pH 7.5) in MaxiSorp microtiter plates (Nunc) overnight. Wells were then washed with PBS-Tween (0.05% (*v*/*v*)) and blocked with 2% BSA in PBS followed by incubation for one hour with galectins diluted in EPBS. Incubation with anti-His_6_-peroxidase (Roche) was done subsequently. Microtiter plates were read out at 495 nm after conversion of OPD substrate (*o*-phenylenediamine, Dako, Hamburg, Germany). Measured data were analyzed using Sigma Plot (Systat software GmbH, Erkrath, Germany).

### 2.7. Inhibition of Galectin Binding with Neo-Glycoproteins

Neo-glycoproteins **13b**, **13d**, **13e** and **14b**, **14d**, **14e**, as well as glycans **1**, **2**, **6** and **7** were used in a competitive inhibition assay [37]. Asialofetuin (ASF, 5 pmol in PBS) as the standard glycoprotein was immobilized overnight in microtiter plates (MaxiSorp, Nunc). After blocking as described before, different concentrations of inhibitor and His_6_Gal-3 were simultaneously incubated for one hour in PBS. Residual bound galectin was detected as described above. Evaluation of the measured data was done using Sigma Plot. All assays were reproduced in at least three independent measurements.

### 2.8. Surface Plasmon Resonance Spectroscopy

SPR spectroscopy was done with Reichert SR7500DC System (XanTec bioanalytics, Düsseldorf, Germany) and carboxymethyldextran hydrogel sensor chips complexed with Ni^2+^ ions (200M, XanTec bioanalytics). These surfaces allow reversible immobilization of His_6_-tagged proteins. Human His_6_Gal-3 (5 pmol in EPBS) was immobilized on the sample channel with a flowrate of 10 µL/min after 5 mM NiCl_2_ solution was injected. The reference channel stayed untreated.

The binding experiments were carried out with a flow rate of 20 µL/min by injecting neo-glycoproteins **13a**–**f** and **14a**–**f** (2 pmol in PBS) and recently synthesized neo-glycoproteins **15a**–**f** and **16a**–**f** [37] (2 pmol in PBS), as well as BSA and ASF as the control. The dissociation time was three minutes. Between the measurements, the surface was regenerated with 0.5 M Na EDTA (pH 8.5) to remove Ni^2+^ together with Gal-3. Each cycle started with NiCl_2_ followed by Gal-3 application.

The measured data were subtracted by reference values and analyzed using Integrated SPRAutolink (Reichert technologies, Depew, NY, USA) and Scrubber2 (version 2.0c, BioLogic Software, Campbell ACT, Australia).

## 3. Results and Discussion

### 3.1. Biotinylation of LacNAc-LacNAc and LacdiNAc-LacNAc

The synthesis of novel glycans and neo-glycoproteins took place with the background of designing new ligands for galectins, especially Gal-3. In this study, the influence of conjugated biotin on galectin binding to tetrasaccharides was investigated. Recently, we could show the benefit of ligand presentation on a protein [37]. We could prove higher affinity of Gal-3 for LacNAc-LacNAc- and LacdiNAc-LacNAc-conjugated BSA with increasing multivalency and compared to free tetrasaccharides. Here, we extended the saccharide part by hydrophobic biotin coupling.

**Scheme 1:**
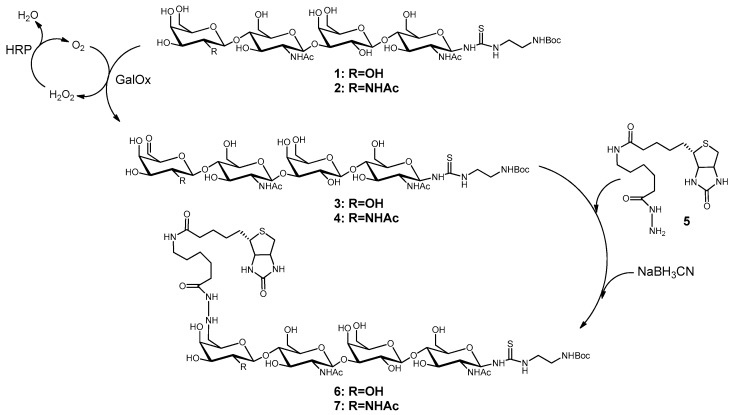
Two-step synthesis of 6-biotin tetrasaccharides **6** and **7**.

The biotinylation of LacNAc oligomers is a two-step synthesis (Scheme 1) as published before [38]. In the first step, the terminal galactose or *N*-acetylgalactosamine (GalNAc) was oxidized at the C6-position by galactose oxidase yielding C6-aldehydes. The second step describes the reaction with BACH (**5**) and subsequent reduction.

The conversion of LacNAc-LacNAc-linker-NH_2_-*t*Boc (**1**) and LacdiNAc-LacNAc-linker-NH_2_-*t*Boc (**2**) to the corresponding C6-aldehydes **3** and **4** by galactose oxidase obtained about 80–90% of product. Stopping the reaction by ultrafiltration avoided the formation to α,β-unsaturated aldehydes as unwanted side-products. Further purification was not necessary. The reaction of **5** with **3** and **4**, respectively, resulted in 6-biotin LacNAc-LacNAc-linker-NH_2_-*t*Boc (**6**) and 6-biotin LacdiNAc-LacNAc-linker-NH_2_-*t*Boc (**7**). After preparative HPLC, yields of 41% (**6**) and 27% (**7**) were obtained. Product loss was probably due to product instability caused by incomplete reduction after oxidation and biotinylation. The isolated products were confirmed by LC-MS (Figure S1A,B). Further product characterization was previously described [38].

### 3.2. Synthesis and Analysis of Neo-Glycoproteins Carrying Biotinylated Glycans

**Scheme 2:**
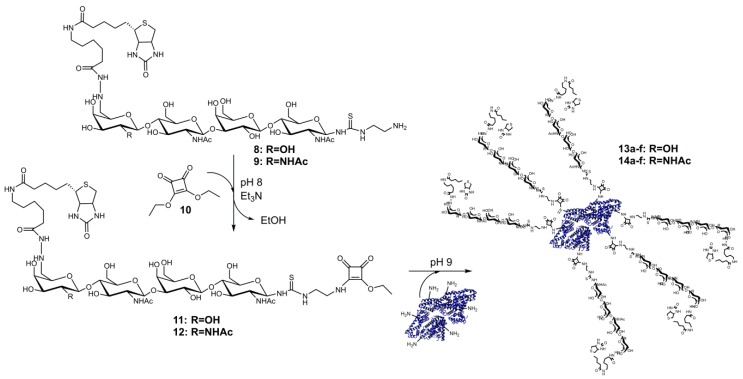
Neo-glycoprotein synthesis by reaction of BSA and squaric acid monoamide ester glycosides **11** and **12**.

Recently, we successfully used squaric acid diethyl ester (**10**) as a bifunctional aminoreactive linker [43] for tunable conjugation of glycans to BSA [37]. In the same way, we synthesized here multivalent BSA-based neo-glycoproteins presenting biotinylated tetrasaccharides (Scheme 2). First, the *t*Boc-linker structures of **6** and **7** were deprotected as described previously [37] to obtain amino-linker functionalized products **8** and **9**. MS-spectra of these compounds are shown in the Supplementary Materials (Figure S1C,D). Since compound **10** reacts also with secondary amines [44], which are present in the biotin moiety, reactions of **8** and **9** were optimized. It turned out that reaction at pH 8.0 and 4 °C for one hour was suitable to produce high amounts of **11** and **12** (Scheme 2). The products were separated from by-products by preparative HPLC, and yields of 42% (2.1 µmol) for **11** and 74% (2.4 µmol) for **12** were obtained. Starting from tetrasaccharides **1** and **2**, overall yields of approximately 15% were obtained. LC-MS results (Figure S1E,F) confirmed the integrity and purity of compounds **11** and **12**.

The products **11** and **12** were further used for glycosylation of BSA. In a modified version of our published procedure [37], a fed-batch addition of compounds **11** and **12**, respectively, was performed. Glycans were added consecutively to the BSA containing reaction mixtures at several time points to overcome squaric acid monoamide ester hydrolysis at pH 9 [43,45]. Finally, different molar ratios of glycans and BSA lysine residues (from 0.03:1 up to 1.2:1) were applied to reach different degrees of glycan modification (Scheme 2).

**Figure 1:**
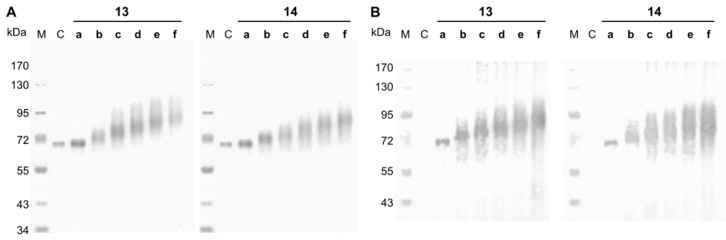
SDS-PAGE and streptavidin blot analysis of neo-glycoproteins. The BSA-based neo-glycoproteins were analyzed by SDS-PAGE with Coomassie staining (**A**) and by a streptavidin blot (**B**); M, Marker; C, control (BSA); **13a**–**f**, 6-biotin LacNAc-LacNAc conjugated BSA; **14a**–**f**, 6-biotin LacdiNAc-LacNAc conjugated BSA.

Neo-glycoproteins were purified by ultrafiltration to remove unbound glycans and analyzed by SDS-PAGE (Figure 1). Coupling of **11** or **12** to BSA led to theoretical increase of BSA protein mass by 1281.5 or 1322.5 g/mol per attached glycan, respectively. Shifts towards higher molecular weights are clearly visible in Figure 1A for neo-glycoproteins **13b**–**f** and **14b**–**f** accompanied by increased broadening of protein bands. The smearing character of the bands of glycosylated BSA resulted from irregular binding of detergents like SDS due to hydrophilic glycans [46] and probably a distribution of coupling degrees within the neo-glycoprotein fractions. Neo-glycoproteins **13b**–**f** and **14b**–**f** show a significant increase of molecular masses with increasing glycosylation densities. Neo-glycoproteins **13a** and **14a** were only modified to a minor degree. In addition, a streptavidin blot analysis proved the biotinylation of the neo-glycoproteins **13a**–**f** and **14a**–**f**, while unmodified BSA was not detectable (Figure 1B).

Molecular mass shifts in SDS-PAGE allow the estimation of achieved coupling degrees of the neo-glycoproteins, which correspond very well to chemical analysis of free lysine residues as previously demonstrated by us [37]. We here determined R_f_ values of protein bands and calculated the attached number of glycans per BSA by molecular weight differences of neo-glycoproteins compared to unmodified BSA (Table 1). The variation of molar excess of glycans with respect to BSA lysine residues gave different degrees of glycan modification of BSA. In general, a slightly lower modification of BSA with 6-biotin-LacdiNAc-LacNAc was observed (**14a**–**f**) when compared to 6-biotin-LacNAc-LacNAc (**13a**–**f**). With the highest applied molar excess (1.2-fold), 14.2 (**13f**) and 11.1 (**14f**) glycans per BSA molecule, respectively, were reached. Coupling efficiencies of 13%–24%, respectively, as the ratio of the amount of attached glycan and the amount of applied glycan are obtained with higher values for 6-biotin-LacNAc-LacNAc. A maximum number of 30 addressable sites of BSA for modification with lactose were previously reported, which is half of the lysine residue amount per BSA molecule [47]. We could confirm that 29 and 28 lysine residues per BSA were modified by the tetrasaccharides LacNAc-LacNAc and LacdiNAc-LacNAc, respectively [37]. Only half of the maximum glycosylation density was achieved for the biotinylated glycans, although the coupling reaction was performed in fed-batch mode. We conclude that limited coupling efficiency is dependent on the molecular mass of the conjugating compound. Biotinylation of the tetrasaccharides increases the molecular masses by 40% and consequently reduces the coupling density by about 50%. This was also recently demonstrated for higher molecular weight biantennary *N*-glycans where a maximal coupling degree of 15 glycans per albumin was reached by click-chemistry addressing lysine residues, despite over a 70-fold molar excess of glycan [48].

In conclusion, novel neo-glycoproteins based on BSA were produced using biotinylated tetrasaccharides as glycans. Glycosylation densities between one and a maximum of 14 lysine residues per BSA molecule were obtained. The biotin-labelled glycans were detected by streptavidin. The impact of biotin-modified oligosaccharides on galectin binding was evaluated in the following binding assays.

### 3.3. Binding of Galectin-3 to Immobilized 6-Biotinylated Tetrasaccharides

**Figure 2:**
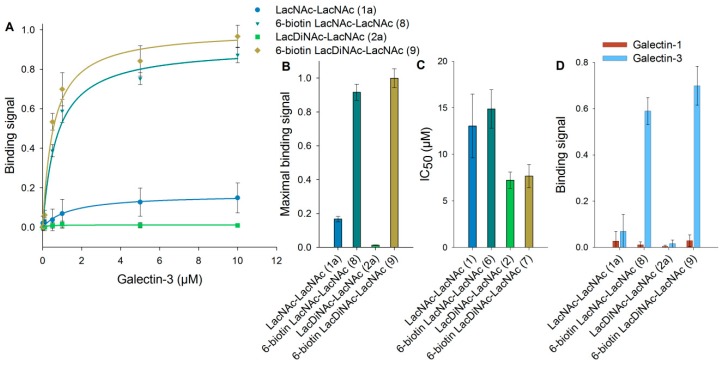
Influence of 6-biotinylation of tetrasaccharides LacNAc-LacNAc and LacdiNAc-LacNAc on galectin binding and inhibition. Binding curves (**A**) and maximal binding signals (**B**) of Gal-3 to immobilized glycans show differences between biotinylated and non-biotinylated ligands. In competitive inhibition assays, the inhibitory potency was not affected by C6-biotinylation of the tetrasaccharides (**C**). Comparison of Gal-3 and Gal-1 at a 1 µM protein concentration indicates high specificity of biotinylated tetrasaccharides towards Gal-3 (**D**).

The biotinylated tetrasaccharides **8** and **9**, as well as the corresponding non-biotinylated and deprotected compounds **1** and **2** (**1a**, **2a**) were immobilized on aminoreactive surfaces to test galectin binding in an ELISA-type assay (Figure 2). Whereas binding to immobilized non-biotinylated tetrasaccharides **1a** and **2a** was weak, Gal-3 showed high binding signals with immobilized biotinylated compounds **8** and **9** (Figure 2A,B). Most interestingly, highest binding was detected for biotinylated LacdiNAc-LacNAc (**9**) and essentially no binding for **2a**. Similarly, Gal-3 binding to LacNAc-LacNAc (**1a**) was rather weak, but increased significantly by C6-biotinylation (**8**). We conclude that binding of Gal-3 to surface immobilized tetrasaccharides is limited by glycan length. However, biotin obviously promotes binding of Gal-3 to surface immobilized tetrasaccharides and could be helpful for boosting binding efficiencies of glycan ligands to Gal-3. The mode of biotin binding to Gal-3 remains unclear. However, our observation of biotin binding of Gal-3 may be related to studies on Gal-3 binding to specific peptide sequences [49].

We further investigated competitive inhibition of galectin-3 binding to ASF by 6-biotinylated tetrasaccharides. Figure 2C shows similar IC_50_ values for biotinylated (**6**, **7**) and non-biotinylated (**1**, **2**) tetrasaccharides. IC_50_ values for **1** and **6** were 14 µM and for **2** and **7** around 8 µM, demonstrating that *N*-acetylation increased inhibition potency by over 40%.

Comparison of Gal-1 and Gal-3 revealed a high selectivity of immobilized 6-biotinylated compounds **8** and **9** for Gal-3 binding (Figure 2D). This is in contrast to previously published data showing that Gal-3 binding is blocked by 6-sialylation of LacNAc glycans [50,51]. Hence, we conclude that 6-biotinylated Lac(di)NAc-LacNAc tetrasaccharides are novel ligands for human galectin-3. Glycan immobilization plays an important role for ligand recognition because the inhibitory potency of soluble glycans was not altered by biotinylation.

### 3.4. Binding of Galectin-3 and Galectin-1 to Neo-Glycoproteins

**Figure 3:**
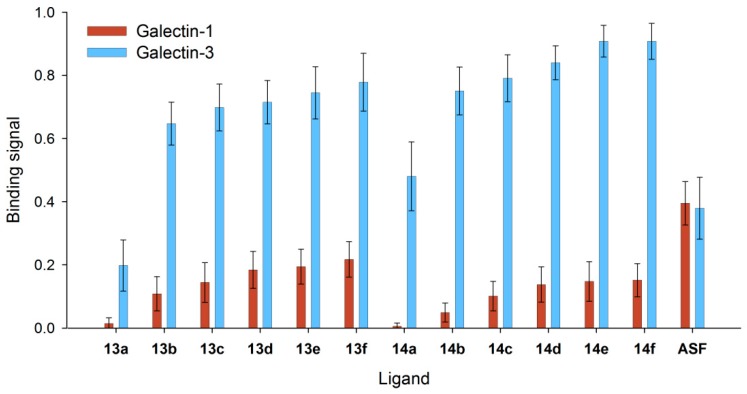
Comparison of Gal-1 and Gal-3 binding to immobilized neo-glycoproteins. In the ELISA-type assay, binding of 1 µM Gal-1 (■) and Gal-3 (■) to neo-glycoproteins **13a**–**f** and **14a**–**f** and to asialofetuin (ASF) as the control was measured. Signals showed different binding signals of both galectins to BSA decorated with C6-biotinylated tetrasaccharides.

Coupling of glycans to proteins is a form of immobilization to achieve multivalent ligand presentation. Since we monitored a significant enhancement of Gal-3 binding to immobilized 6-biotin tetrasaccharides, we tested BSA-based neo-glycoproteins carrying either 6-biotin LacNAc-LacNAc (**13a**–**f**) or 6-biotin LacdiNAc-LacNAc (**14a**–**f**). Neo-glycoproteins were immobilized for the evaluation of Gal-3 and Gal-1 binding in a solid-phase assay. Comparison was made with Gal-1 as a member of the large prototype family. In terms of specificity and selectivity, we expect similar results for other members of the prototype family because glycan ligand specificity is similar among the members of this family. Figure 3 demonstrates that Gal-3 binds very efficiently to neo-glycoproteins compared to Gal-1. As a control, both galectins show identical binding to ASF.

For all BSA-based neo-glycoproteins varying in glycosylation densities, Gal-3 binding signals were more than four-fold higher than Gal-1 binding signals (see also Table S1). The binding differences were more pronounced with LacdiNAc glycans (**14a**–**f**). Moreover, specificity for Gal-3 was more pronounced at a lower degree of glycan modification with less than three glycans per BSA molecule (**13a**, **14a**,**b**). Gal-3 binds to **13a** and **14b** 15-times better and to **14a** even 80-times better than Gal-1. In contrast, at higher modification degrees, Gal-3 binding signals were on average five-fold higher than those for Gal-1.

The galectin binding signals for neo-glycoproteins presenting biotinylated glycans were similar to those obtained in our previous study for non-biotinylated tetrasaccharide-conjugated BSA [37]. However, at least two-times higher Gal-3 binding signals are already reached for neo-glycoproteins presenting at least two biotinylated glycans. For the non-biotinylated counterparts, more than six glycans per BSA molecule were needed. To reach 75% of the maximum binding signal, twice the number of LacNAc-LacNAc glycans compared to 6-biotin LacNAc-LacNAc glycans and six-fold more of LacdiNAc-LacNAc glycans compared to 6-biotin LacdiNAc-LacNAc should be presented on one BSA molecule (Table S2). Although Gal-1 should not be able to bind internal galactose [52,53], Gal-1 bound weakly to 6-biotinylated LacNAc-LacNAc as well as LacdiNAc-LacNAc (Figure 3). Since modifications at the C6-position like sulfation or sialylation and at the C2-position like acetylation are not tolerated by Gal-1 [50,51,54,55], binding signals of Gal-1 to 6-biotinylated tetrasaccharides are probably caused by weak recognition of internal galactose [37,41,56,57] when multiple ligands are presented.

We conclude that in the case of 6-biotinylated tetrasaccharides, preferably few glycans presented on BSA trigger high Gal-3 binding. These signals were higher compared to non-biotinylated neo-glycoproteins as reported in our recent study [37]. Moreover, statistically one or two 6-biotinylated LacdiNAc-LacNAc glycans per BSA molecule (**14a**,**b**) are sufficient to achieve almost absolute selectivity for Gal-3 binding over Gal-1. Since Gal-3 shows already exceptional binding to neo-glycoproteins **14a**–**c** presenting up to four glycans, the multivalency of biotinylated glycans on neo-glycoproteins seems to play a minor role and does not amplify galectin binding.

### 3.5. Detailed Characterization of Galectin-3 Binding to Neo-Glycoproteins

With neo-glycoproteins carrying LacNAc- and LacdiNAc-terminated tetrasaccharides, multivalency was the key for the detection of Gal-3 concentrations below 0.05 µM [37]. Therefore, we investigated Gal-3 binding to 6-biotin LacNAc- and 6-biotin LacdiNAc-terminated tetrasaccharides at varying Gal-3 concentrations.

**Figure 4:**
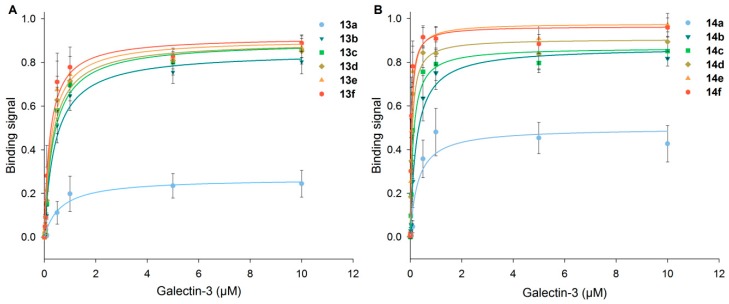
Binding of human Gal-3 to neo-glycoproteins presenting different numbers of glycans. In an ELISA-type assay, Gal-3 at different concentrations was incubated on immobilized 6-biotin LacNAc-LacNAc- (**13a**–**f**) (**A**) and 6-biotin LacdiNAc-LacNAc-conjugated (**14a**–**f**) BSA (**B**) (5 pmol per well). Binding signals were plotted against galectin concentration and fitted.

Binding signals of Gal-3 on immobilized neo-glycoproteins **13a**–**f** and **14a**–**f** increased with higher Gal-3 concentration as well as higher glycan densities (Figure 4). A significant difference in Gal-3 binding was monitored for those neo-glycoproteins with minor modification degrees. However, with two or more glycans, only a minor increase of galectin binding was observed, being more pronounced for 6-biotin LacdiNAc-LacNAc-conjugated BSA (**14a**–**f**). With **14a**–**f**, overall higher binding signals of Gal-3 were reached compared to **13a**–**f**. It is known that the hydroxyl group at the C2-position is not involved in interacting with the CRD of Gal-3 [58]. Thus, C2-modification is tolerated by Gal-3 and even enhances galectin binding to the LacdiNAc epitope, as reported earlier [29,33,37,54]. However, modifications at the C6-position of the galactoside are not tolerated in the case of Gal-3 binding because hydrogen bond formation is obligatory for the binding process [54,59,60]. In our study, we demonstrate the positive effect of BACH conjugated to the non-reducing Lac(di)NAc unit of the corresponding tetrasaccharides. The spacer at the C6-position used in this study allows even the formation of two hydrogen bonds. Potentially, structural modelling data of Gal-3 for binding Lacto-*N*-neo-tetraose (LNnT) could be interpreted that C6-modification of the non-reducing LacNAc unit may be tolerated [61]. Thus, it is possible to design Gal-3 ligands that carry functionalities that do not interact directly with the binding sites, but with more distant residues of Gal-3. The conjugation of the tetrasaccharides with biotin could also enhance the Gal-3 binding to the internal LacNAc unit by optimizing the ligand protein complex regarding orientation. With respect to effective hydrophobic modifications at the C3-position [62,63,64,65], the C6-biotinylated non-reducing terminal LacNAc and LacdiNAc structures may be considered as sophisticated glycan-derived hydrophobic C3-modifications of LacNAc. The precise role of the C6-biotin modification and its interaction with the binding domain of Gal-3 remains a matter of protein modelling studies.

**Figure 5:**
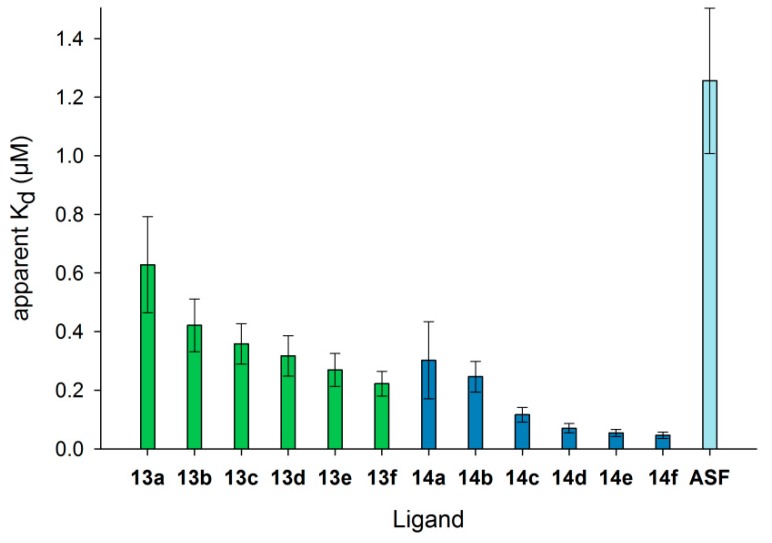
Apparent K_d_ values of Gal-3 for binding to immobilized neo-glycoproteins. The Gal-3 concentration (µM) at half-maximal binding (K_d_) is shown for the different neo-glycoproteins **13a**–**f** (■) and **14a**–**f** (■), as well as ASF (■). K_d_ values decreased with increasing modification degree, indicating higher affinity of human Gal-3 (see Table S3 in the Supplementary Materials).

Differences in Gal-3 binding to 6-biotin LacNAc-LacNAc- and 6-biotin LacdiNAc-LacNAc-conjugated BSA are elucidated when the apparent K_d_ data are compared (Figure 5, Table S3, Supplementary Materials). The K_d_ value is the concentration at which 50% saturation of the binding signal is reached and a measure for binding affinity. Gal-3 depicts the highest affinity (lowest apparent K_d_ values) for neo-glycoproteins **14a**–**f** (6-biotin LacdiNAc-LacNAc) compared to neo-glycoproteins **13a**–**f** (6-biotin LacNAc-LacNAc). Most interestingly, ASF having nine N-glycosidic bound LacNAc units is not the preferred glycoprotein (highest apparent K_d_). Gal-3 was characterized to bind to the first LacNAc epitope of the triantennary N-glycan of ASF with high affinity that is reduced for the other epitopes due to increasing negative cooperativity [66]. Additionally, a longer oligosaccharide consisting of at least two LacNAc units was proven to be bound by Gal-3 with higher affinity than one LacNAc unit [41]. The apparent K_d_ values for **13a**–**f** decrease gradually and reach 0.22 µM for the highest modification degree of 14 glycans per BSA molecule (**13f**) (Table S3, Supplementary Materials). The values for **14a**–**f** are overall lower with a significant drop between two (**14b**) and four (**14c**) glycans per molecule. For neo-glycoprotein **14f** with 10.5 glycans per BSA molecule, Gal-3 shows the highest affinity (K_d_ 0.05 µM). Despite the lower glycosylation density of **14f** compared to **13f**, the K_d_ value was more than four-fold lower, emphasizing the positive influence of the *N*-acetylated galactosamine. In comparison to BSA presenting non-biotinylated tetrasaccharides [37], Gal-3 affinity for **13a**–**f** and **14a**–**f** is higher than for the corresponding non-biotinylated neo-glycoproteins with a similar glycosylation level.

With respect to the minimum number of glycans to obtain high Gal-3 affinity, biotinylated glycan ligands are superior over non-biotinylated glycans. A K_d_ value below 0.1 µM was already reached for 6-biotin LacdiNAc-LacNAc with a glycosylation density of six glycans per BSA molecule (**14d**) compared to 14 LacdiNAc-LacNAc glycans per BSA molecule [37], whereas neo-glycoproteins presenting LacNAc-LacNAc did not achieve this K_d_ value. We conclude that terminal GalNAc improved the affinity of Gal-3, and additional 6-biotinylation gained a further affinity increase.

The binding strength of a lectin can be drastically increased by multivalent ligand presentation due to glyco clusters [66,67,68,69,70]. Gal-3 forms pentamers or oligomers during binding events [26,71], and multivalent carbohydrate presentation plays an important role in binding enhancement [66,72,73,74]. We recently demonstrated enhanced avidity of Gal-3 for multivalent presentation of LacdiNAc-terminated tetrasaccharides as neo-glycoproteins based on BSA [37]. Up to 100-fold binding enhancement was reached with LacdiNAc-LacNAc-conjugated BSA modified with at least 21 glycans at a Gal-3 concentration of 5 nM.

Since C6-biotinylation of the conjugated tetrasaccharides triggers the Gal-3 binding, a multivalent effect may also be involved. Converting K_d_ values into binding potencies relative to ASF and calculating the relative potency per glycan (Table S3, Supplementary Materials), binding enhancement due to multivalency was, however, not observed. Even neo-glycoproteins with low number of glycans are high affinity ligands for Gal-3, especially 6-biotin LacdiNAc-LacNAc-conjugated BSA, so that more glycans on one BSA molecule had hardly any influence. Moreover, the maximal achieved glycan density is probably too low to gain enhanced Gal-3 binding. Eight conjugated glycans to BSA-based neo-glycoprotein were reported to be too distant to reach enhanced avidity for Gal-1 [52].

### 3.6. Neo-Glycoproteins as Inhibitors for Galectin-3

High-affinity glycan ligands for Gal-3 are of interest as inhibitors in biomedical applications [51,75,76]. In this context, we performed inhibition assays for Gal-3 binding to immobilized ASF using neo-glycoproteins of low (**13b**, **14b**), medium (**13d**, **14d**) and high modification degrees (**13e**, **14e**).

**Figure 6:**
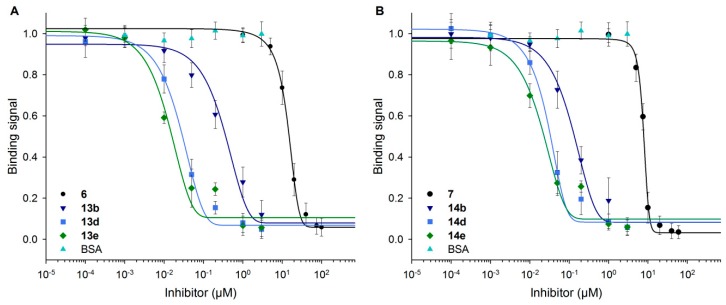
Inhibition of Gal-3 binding to ASF by BSA-based neo-glycoproteins. With increasing concentrations of 6-biotin LacNAc-LacNAc-conjugated (**A**) and 6-biotin LacdiNAc-LacNAc-conjugated BSA (**B**) as well as monovalent controls (**6** and **7**), Gal-3 binding signals on ASF decreased. The curves shifted to lower inhibitor concentrations with rising glycosylation density.

Inhibition curves in Figure 6 indicate the decrease of the Gal-3 signal with increasing inhibitor concentrations for neo-glycoproteins and free glycans (**6**, **7**). BSA as the control showed no inhibitory effect. As stronger inhibition is shown by a curve shift to lower inhibitor concentrations, neo-glycoproteins showed higher inhibition potencies with increasing glycosylation densities than free tetrasaccharides. A significant shift occurs already for BSA-based neo-glycoproteins decorated with at least two glycans (**13b**, **14b**). The effect of increased glycan density was less pronounced, except for the difference of **13b** and **13d**.

**Figure 7:**
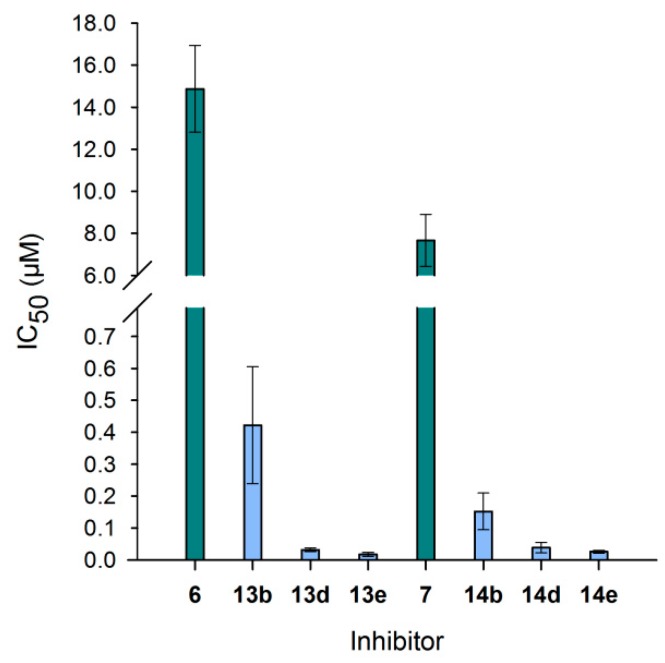
IC_50_ values of neo-glycoproteins for Gal-3 inhibition. IC_50_ values of selected neo-glycoproteins (■) and the corresponding free saccharides (■) are compared. Neo-glycoproteins showed higher inhibitory potential compared to monovalent glycans, as well as with increasing number of glycans per molecule. Calculated IC_50_ values are depicted in Table 2.

IC_50_ values were calculated and showed clear differences between the inhibitory potencies of neo-glycoproteins and corresponding free glycans (Figure 7 and Table 2). With BSA presenting three 6-biotin LacNAc-LacNAc glycans (**13b**), a 35-fold increase of inhibition potency was reached compared to the free biotinylated tetrasaccharide **6**. Presenting additional six glycans (**13d**), the IC_50_ value is lowered by a factor of 14 compared to **13b**, reaching a low nanomolar concentration. A further increase in the number of conjugated glycans does not affect inhibition potencies. For 6-biotin LacdiNAc-LacNAc-modified BSA with two glycans (**14b**), a 50-fold higher inhibitory potency is gained compared to the free 6-biotin LacdiNAc presenting tetrasaccharide **7**, being more potent than **6**. The presentation of more glycans on BSA (**14d**, **14e**) improved the inhibition potential merely by a factor of about four. Both biotinylated glycan types with medium to high BSA modification degrees (**13d**, **13e** and **14d**, **14e**) achieved IC_50_ values in the low nanomolar range.

Presentation of 6-biotinylated tetrasaccharides as neo-glycoprotein affected Gal-3 binding positively when compared to non-biotinylated counterparts of our recent study [37]. As C6-biotinylation of the tetrasaccharides caused no improvement using them as soluble ligands in an inhibition assay (Figure 2C), the application of neo-glycoproteins is beneficial for binding characterization, as well as the design of high-affinity ligands.

Contrary to the Gal-3 binding assay on immobilized neo-glycoproteins, multivalent presentation of the glycans had a relevant impact on Gal-3 inhibition by neo-glycoproteins in solution. Relative potencies calculated per glycan reveal multivalency effects (Table 2). Regarding 6-biotin LacNAc-LacNAc, neo-glycoprotein **13b** (three glycans) showed eleven-fold, **13d** (nine glycans) 50-fold and **13e** (13 glycans) almost 70-fold inhibitory potency in relation to one binding site compared to **6**. The multivalent influence for 6-biotin LacdiNAc-LacNAc-conjugated BSA was less pronounced. Relative inhibitory potencies of 24–34 per glycan were observed for **14b**, **14d** and **14e** compared to **7**. The overall lower potencies are due to the already higher inhibition potential of **7** compared to **6**. However, **14b** showed higher potency of one binding site than **13b**. Consequently, higher modification degrees than two glycans per BSA (**14d**, **14e**) did not achieve elevated potencies. The increase of Gal-3 avidity is known to be affected if multivalent ligands are presented due to the formation of oligomeric Gal-3 clusters [26,71]. Multivalent dendrimers and calixarenes modified with lactose revealed strong multivalent effects in a solid phase inhibition assay with ASF [77,78]. An IC_50_ of 200 µM was received with lactose presenting calixarenes that represented a 12.5-fold improved inhibitory potency [78]. Calix[4/6]arenes mutivalently presenting LacNAc were shown to lower the IC_50_ value by a factor of 1500 to 0.15 µM [79]. We could obtain IC_50_ values in the nanomolar range even with conjugation densities below ten for 6-biotinylated tetrasaccharides on BSA. In conclusion, biotinylation of the BSA-conjugated Lac(Di)NAc terminated tetrasaccharides showed high inhibitory potencies at low glycan densities.

### 3.7. Neo-Glycoproteins as Galectin-3 Ligands in Surface Plasmon Resonance Spectroscopy

Albumin modified with suitable glycans is of high interest for the design of galectin ligands, as the present and recent studies show [37,48,52]. We could already prove Gal-3 binding to neo-glycoproteins in a solid phase assay. SPR spectroscopy was additionally performed to compare neo-glycoproteins as Gal-3 ligands in a flow setup. Gal-3 was immobilized via His_6_-tag on a Ni^2+^-dextran surface, and binding of neo-glycoproteins as well as ASF was monitored. This setup allowed complete removal of Gal-3 by releasing of Ni^2+^ by EDTA solution. Thus, strong interactions of Gal-3 and ligand had no influence on the following measurement because fresh Gal-3 solution was used for each ligand.

The neo-glycoproteins designed in this study as well as selected neo-glycoproteins of our recent study were compared [37]. Four different tetrasaccharides, 6-biotin LacNAc-LacNAc (**13a**–**f**), 6-biotin LacdiNAc-LacNAc (**14a**–**f**), LacNAc-LacNAc (**15a**–**f**) and LacdiNAc-LacNAc (**16a**–**f**), were presented on BSA with different modification degrees (Table 3).

**Figure 8:**
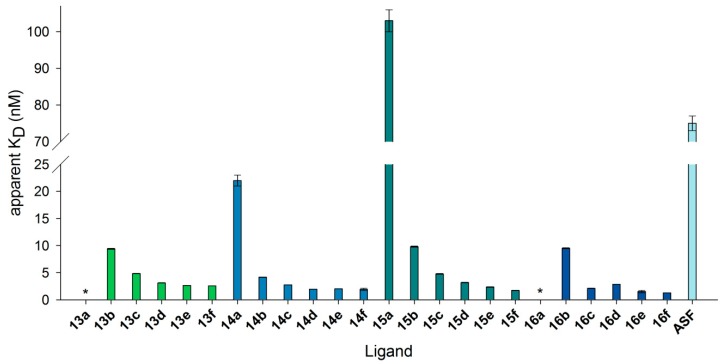
K_D_ values resulting from binding of neo-glycoproteins on immobilized Gal-3 in SPR measurements. Gal-3 was immobilized on a sensor chip, and binding to neo-glycoproteins as well as ASF was monitored in flow (20 µL/min). Apparent K_D_ values were calculated for one concentration by fitting association and dissociation using the software Scrubber2. The numbering of the ligands is explained in Table 3. (* K_D_ was not detectable).

Figure 8 reveals that biotinylated glycans gave low dissociation constants (K_D_) at lower modification densities. K_D_ is defined as the ratio of k_off_ and k_on_ and therefore calculated by fitting dissociation and association curves. We observed typical sensorgrams with a clear association and dissociation phase as previously published for a similar setup [48,73] (Figure S2, Supplementary Materials). The SPR sensorgrams show steeper slopes for association with increasing modification degree of neo-glycoproteins. The dissociation was very slow with a relative high response level indicating that glycoproteins remained bound to Gal-3 for the tested dissociation time.

All neo-glycoproteins, except **15a** as well as **13a** and **16a** with no calculable values, showed higher binding affinity towards Gal-3 than ASF. Moreover, apparent K_D_ values decreased with increasing glycosylation density, but were mostly in the range of 1–10 nM (see Table S4, Supplementary Materials). No significant increase in affinity caused by increasing multivalency was observed. At a similar modification degree between six and seven (**13c**, **14d**, **15b**, **16b**), BSA presenting biotinylated tetrasaccharides gained lower apparent K_D_ values that could be again reduced for the GalNAc-containing glycans (**14d**). Overall, compared to ASF, up to a 60-fold better binding of neo-glycoproteins to immobilized Gal-3 was observed. This result keeps up with LacNAc-quantum dots presenting 108 LacNAc epitopes and showing 92-fold better binding to Gal-3 compared to free LacNAc [73]. Exactly the same affinity enhancement compared to ASF was described for neo-glycoproteins carrying 15 biantennary *N*-glycans [48]. In summary, with increasing glycosylation density, Gal-3 binds more strongly to non-biotinylated neo-glycoproteins. At low to medium modification degrees, biotinylated conjugated tetrasaccharides led to higher Gal-3 affinity compared to non-biotinylated glycans.

In conclusion, the results of SPR measurements show dissociation constants (K_D_) of biotinylated Lac(di)NA-conjugated BSA-based neo-glycoproteins in the low nanomolar range, which are in agreement with calculated apparent K_d_ and IC_50_ values determined by solid-phase ELISA assays.

## 4. Conclusions

Here, we report for the first time on biotin modification of glycans for the design of selective high-affinity Gal-3 ligands. Tetrasaccharides with C6-biotinylation of the terminal Galactose/GalNAc sugar unit were synthesized and characterized as Gal-3 ligands. Gal-3 binding to immobilized biotinylated glycans was improved in comparison to non-biotinylated counterparts and even enabled high selectivity for Gal-3 when compared to Gal-1. With the design of neo-glycoproteins based on BSA, biotinylated ligands show high affinity and high inhibitory potency even at low glycosylation density. K_d_ and IC_50_ values below 50 nM are obtained.

We conclude that our biotinylated neo-glycoproteins are promising candidates for targeting Gal-3 in cancer-related biomedical research. In addition, the neo-glycoproteins can be further loaded with cytotoxic compounds and fluorescent dyes to yield tailor-made theranostics. Further application could be the capture of Gal-3 from serum of cancer patients on suitable surfaces of biosensors. In conclusion, our findings open new possibilities for C6-modifications of carbohydrate structures in galectin inhibitor design.

## Figures and Tables

**Table 1:** Glycosylation densities of neo-glycoproteins **13a**–**f** and **14a**–**f**. The differences in molecular weights (ΔMW) compared to BSA determined by SDS-PAGE were used to calculate the number of attached 6-biotin LacNAc-LacNAc (**13a**–**f**) and 6-biotin LacdiNAc-LacNAc (**14a**–**f**). Consequential molecular weights (MW) are given, as well.

Compound	MW (kDa)	ΔMW (kDa)	Modified Lysine Residues (mol/mol BSA)
**BSA**	66.4	0.0	0.0
**13a**	67.0	0.6	0.5
**13b**	70.6	4.2	3.3
**13c**	74.6	8.2	6.4
**13d**	78.4	12.0	9.4
**13e**	83.0	16.6	13.0
**13f**	84.6	18.2	14.2
**14a**	66.8	0.4	0.3
**14b**	69.3	2.9	2.3
**14c**	71.9	5.5	4.3
**14d**	74.3	7.9	6.2
**14e**	78.1	11.7	9.2
**14f**	80.6	14.2	11.1

**Table 2:** Calculated IC_50_ values and relative inhibitory potencies of neo-glycoproteins. Values of inhibitory potentials are stated for tested tetrasaccharides and neo-glycoproteins. In the right column, inhibitory potencies were calculated per glycan to identify a possible influence of multivalency.

Compound	IC_50_ (µM)	Relative Potency	Relative Potency per Glycan
**6**	14.8 ± 2.06	1.0 ± 0.1	1.0 ± 0.1
**13b**	0.42 ± 0.18	35.2 ± 15.3	10.7 ± 4.6
**13d**	0.03 ± 0.01	464.1 ± 83.7	49.4 ± 8.9
**13e**	0.02 ± 0.01	856.0 ± 287.5	65.8 ± 22.1
**7**	7.66 ± 1.24	1.0 ± 0.2	1.0 ± 0.2
**14b**	0.15 ± 0.06	50.3 ± 19.1	21.9 ± 8.3
**14d**	0.04 ± 0.02	197.6 ± 84.8	31.9 ± 13.7
**14e**	0.03 ± 0.00	297.9 ± 52.4	32.4 ± 5.7

**Table 3:** Neo-glycoproteins used in SPR measurements. Biotinylated and non-biotinylated LacNAc- and LacdiNAc-terminated tetrasaccharides conjugated to BSA were used as Gal-3 ligands in SPR measurements. The numbering and modification degree of each neo-glycoprotein are shown.

6-Biotin LacNAc-LacNAc		6-Biotin LacdiNAc-LacNAc		LacNAc-LacNAc		LacdiNAc-LacNAc	
**13a**	0.5	**14a**	0.3	**15a**	1.6	**16a**	1.7
**13b**	3.3	**14b**	2.3	**15b**	7.5	**16b**	7.5
**13c**	6.4	**14c**	4.3	**15c**	14.4	**16c**	14.1
**13d**	9.4	**14d**	6.2	**15d**	17.8	**16d**	18.0
**13e**	13.0	**14e**	9.2	**15e**	24.2	**16e**	24.4
**13f**	14.2	**14f**	11.1	**15f**	29.0	**16f**	27.5

## Supplementary Materials

The following are available online at http://www.mdpi.com/2306-5354/4/2/31/s1. Figure S1: MS spectra of products **6**, **7**, **8**, **9**, **11** and **12**; Figure S2: SPR sensorgrams of neo-glycoproteins bound by immobilized galectin-3; Table S1: Binding signals of galectin-1 and galectin-3 to immobilized neo-glycoproteins **13a**–**f** and **14a**–**f** and asialofetuin (ASF); Table S2: Required glycan number attached to neo-glycoproteins to reach 75% of maximum galectin-3 binding; Table S3: K_d_ values and relative potencies of galectin-3 bound to neo-glycoproteins **13a**–**f** and **14a**–**f** and ASF; Table S4: Values of K_D_ in SPR measurements with neo-glycoproteins and immobilized galectin-3.
