# Supplementary material for: Biotinylated N-Acetyllactosamine- and N,N-Diacetyllactosamine-Based Oligosaccharides as Novel Ligands for Human Galectin-3
Source: Bioengineering (Basel). 2017 Apr 5;4(2):31. doi: 10.3390/bioengineering4020031 (PMC5590477; doi:10.3390/bioengineering4020031)
Supplement: Supplementary file 1 [file bioengineering-04-00031-s001.pdf]

bioengineering

MDPI

Supplementary data

Biotinylated *N*-Acetyllactosamine and *N*,*N*-Diacetyllactosamine based Oligosaccharides as Novel Ligands for Human Galectin-3

Sophia Böcker [1] and Lothar Elling [1,*]

[1] Laboratory for Biomaterials, Institute for Biotechnology and Helmholtz-Institute for Biomedical Engineering, RWTH Aachen University, Pauwelsstrasse 20, 52074 Aachen, Germany; s.boecker@biotec.rwth-aachen.de; l.elling@biotec.rwth-aachen.de

* Correspondence: l.elling@biotec.rwth-aachen.de; Tel.: +49-241-8028351

1. Product characterization by LC-MS analysis

A

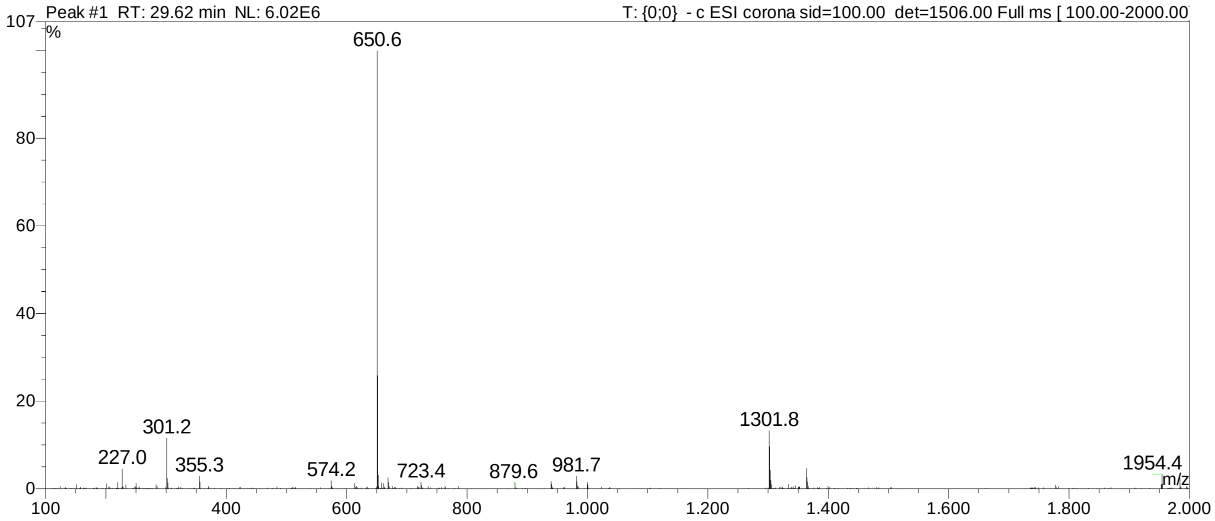

B

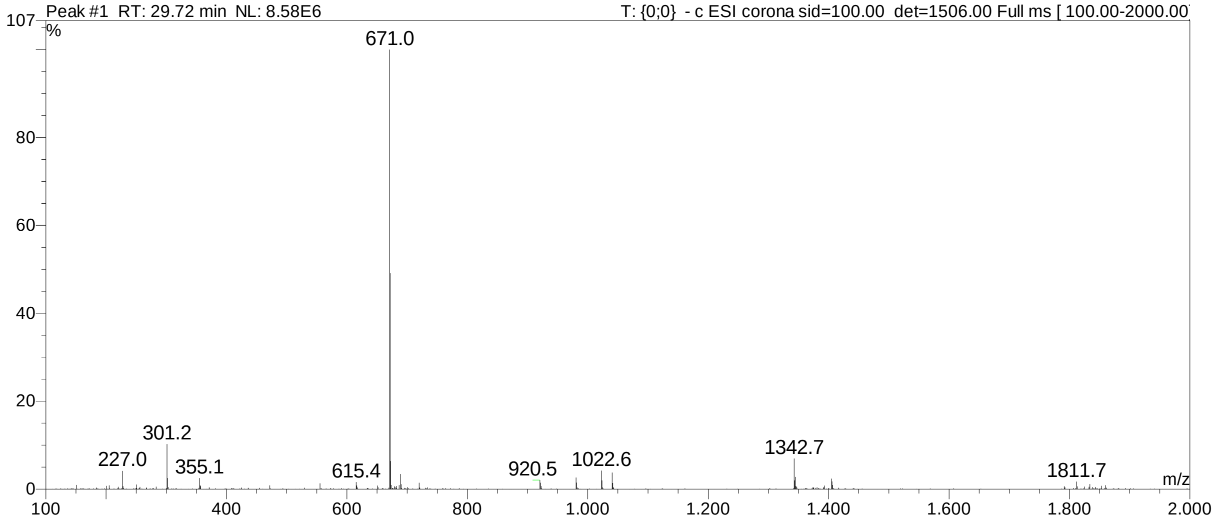

C

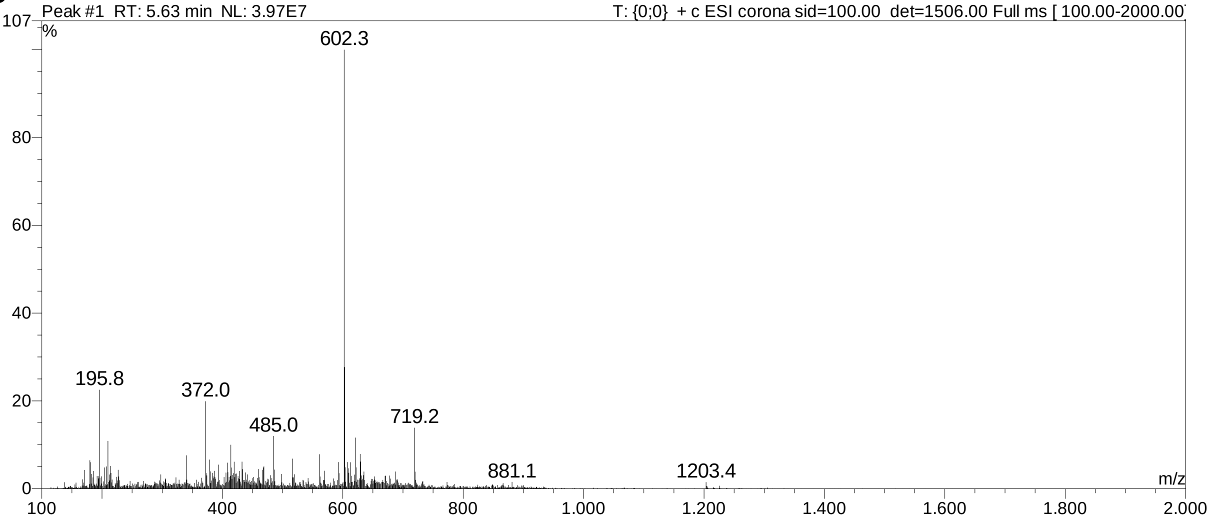
Peak #1 RT: 5.63 min NL: 3.97E7
T: {0;0} + c ESI corona sid=100.00 det=1506.00 Full ms [100.00-2000.00]
%
602.3
195.8
372.0
485.0
719.2
881.1
1203.4
m/z
107
80
60
40
20
0
100
400
600
800
1.000
1.200
1.400
1.600
1.800
2.000

D

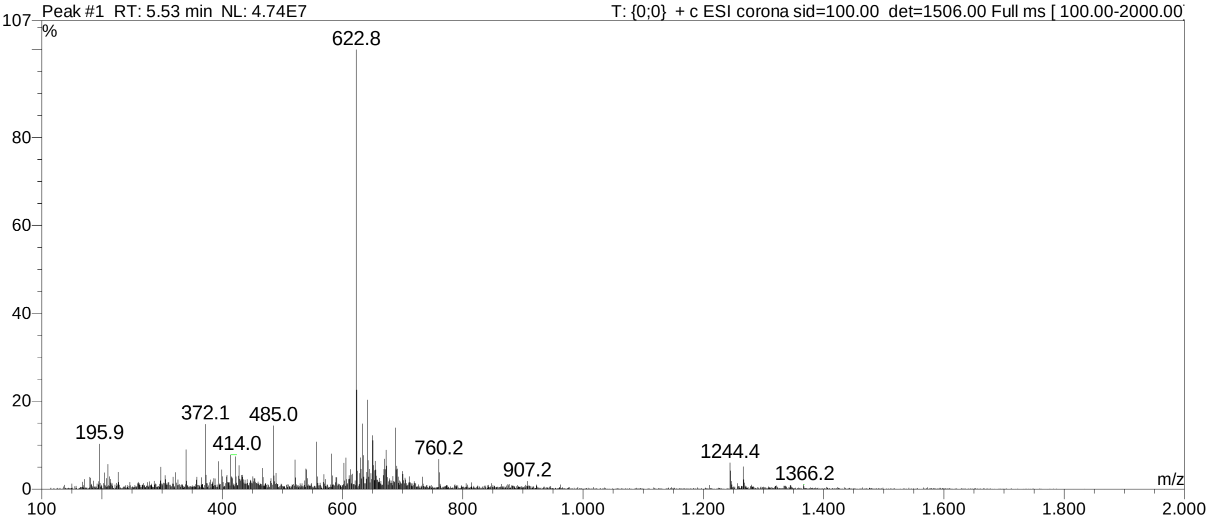
Peak #1 RT: 5.53 min NL: 4.74E7
T: {0;0} + c ESI corona sid=100.00 det=1506.00 Full ms [100.00-2000.00]
%
622.8
195.9
372.1
414.0
485.0
760.2
907.2
1244.4
1366.2
m/z
107
80
60
40
20
0
100
400
600
800
1.000
1.200
1.400
1.600
1.800
2.000

E

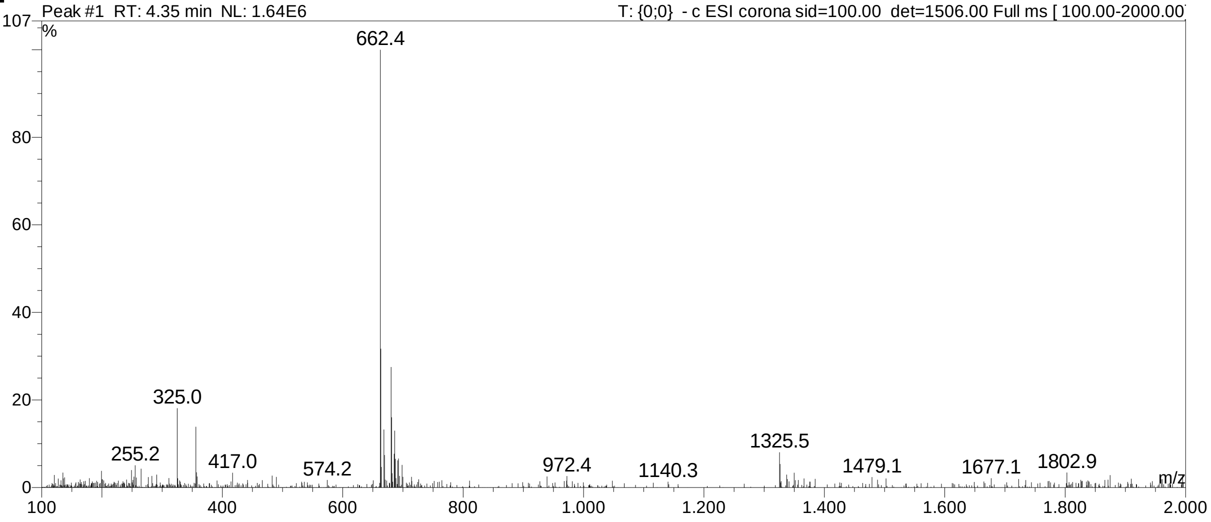
Peak #1 RT: 4.35 min NL: 1.64E6
T: {0;0} - c ESI corona sid=100.00 det=1506.00 Full ms [100.00-2000.00]
%
662.4
255.2
325.0
417.0
574.2
972.4
1140.3
1325.5
1479.1
1677.1
1802.9
m/z
107
80
60
40
20
0
100
400
600
800
1.000
1.200
1.400
1.600
1.800
2.000

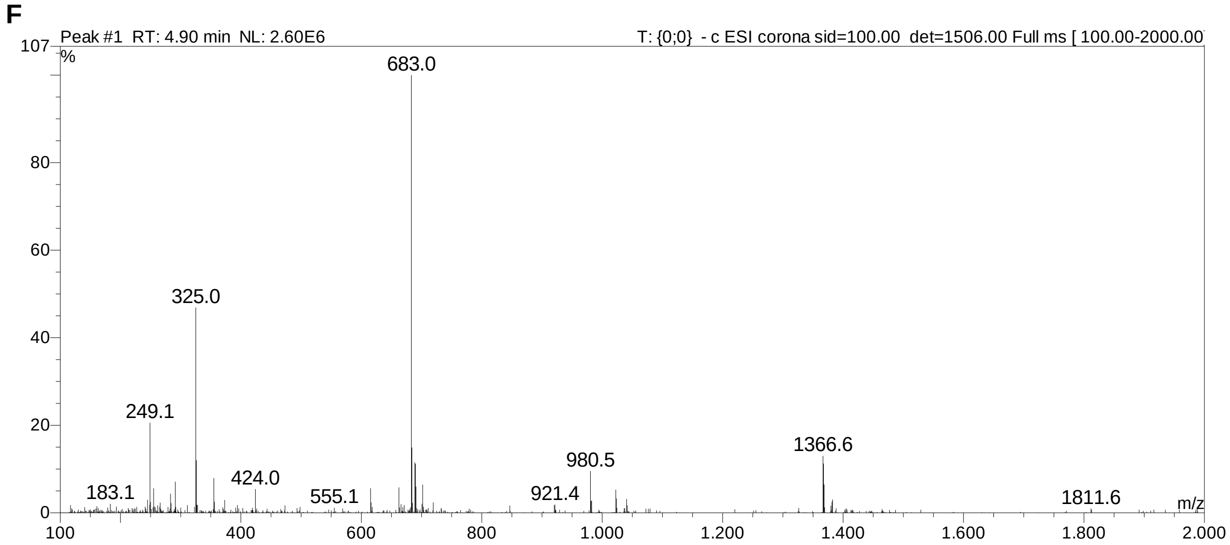

Figure S1. MS spectra of products **6** (**A**), **7** (**B**), **8** (**C**), **9** (**D**), **11** (**E**) and **12** (**F**)

LC-MS analysis using electrospray ionization. [M-H]- ions were analyzed using quadrupole mass analyzer.

6 $[M-H]^-$ = 1301.8 *m/z* ($[M-2H]^{2-}$ = 650.6 *m/z*); calculated *m/z*: 1302.6

7 $[M-H]^-$ = 1342.7 *m/z* ($[M-2H]^{2-}$ = 671.0 *m/z*); calculated *m/z*: 1343.6

8 $[M+H]^+$ = 1204.4 *m*/z ($[M+2H]^{2+}$ = 602.3 *m/z*); calculated *m/z*: 1202.5

9 $[M+H]^+$ = 1244.4 *m/z* ($[M+2H]^{2+}$ = 622.8 m/z); calculated *m/z*: 1243.5

11 $[M-H]^-$ = 1325.5 *m/z* ($[M-2H]^{2-}$ = 662.4 *m/z*); calculated *m/z*: 1326.5

12 $[M-H]^-$ = 1366.6 *m/z* ($[M-2H]^{2-}$ = 683.0 m/z); calculated *m/z*: 1367.6

2. Galectin binding to neo-glycoproteins

Table S1. Binding signals of galectin-1 and galectin-3 to immobilized neo-glycoproteins **13a-f** and **14a-f** and asialofetuin (ASF)

Comparison of galectin-1 and galectin-3 binding is shown with standard deviation of at least 9 measured values. Significant higher binding of galectin-3 compared to galectin-1 is observed for neo-glycoproteins presenting 6-biotin LacNAc-LacNAc and 6-biotin LacDiNAc-LacNAc, respectively.

	**Binding signal**			**Binding signal**	
**Ligand**	**Galectin-1**	**Galectin-3**	**Ligand**	**Galectin-1**	**Galectin-3**
**13a**	0.01 ± 0.02	0.20 ± 0.08	**14a**	0.01 ± 0.01	0.48 ± 0.11
**13b**	0.11 ± 0.05	0.65 ± 0.07	**14b**	0.05 ± 0.03	0.75 ± 0.08
**13c**	0.14 ± 0.06	0.70 ± 0.07	**14c**	0.10 ± 0.05	0.79 ± 0.07
**13d**	0.18 ± 0.06	0.72 ± 0.07	**14d**	0.14 ± 0.06	0.84 ± 0.05
**13e**	0.19 ± 0.06	0.74 ± 0.08	**14e**	0.15 ± 0.06	0.91 ± 0.05
**13f**	0.22 ± 0.06	0.78 ± 0.09	**14f**	0.15 ± 0.05	0.91 ± 0.06
			ASF	0.40 ± 0.07	0.38 ± 0.10

Table S2. Required glycan number attached to neo-glycoproteins to reach 75% of maximum galectin-3 binding

To reach 75% of maximum galectin-3 binding twice of LacNAc-LacNAc and six-fold more LacDiNAc glycans have to be conjugated to BSA compared to the 6-biotinylated counterparts. The neo-glycoproteins presenting non-biotinylated tetrasaccharides were characterized in our recent study [37].

6-biotin LacNAc-LacNAc	6-biotin LacDiNAc-LacNAc	LacNAc-LacNAc	LacDiNAc-LacNAc
13.0	2.3	24.2	14.1

Table S3. K_d values and relative potencies of galectin-3 bound to neo-glycoproteins **13a-f** and **14a-f** and ASF Apparent K_d in [µM] galectin-3 in ELISA-type binding assay to immobilized neo-glycoproteins (5 pmol) and respective standard deviations of at least 9 measured data are shown. Values were calculated by data fitting using equation for one site saturation ($y = \frac{B_{max} \cdot x}{K_d + x}$). Potencies were calculated in relation to ASF and additionally per glycan. Binding affinity of galectin-3 increases with increasing modification densities of neo-glycoproteins, more pronounced for 6-biotin LacDiNAc-LacNAc conjugated BSA (**14a-f**).

Ligand	Apparent K_d [µM]	Relative potency	Relative potency per glycan
**13a**	0.63 ± 0.16	2.00 ± 0.52	4.14 ± 1.08
**13b**	0.42 ± 0.09	2.98 ± 0.64	0.91 ± 0.20
**13c**	0.36 ± 0.07	3.51 ± 0.68	0.55 ± 0.11
**13d**	0.32 ± 0.07	3.96 ± 0.86	0.42 ± 0.09
**13e**	0.27 ± 0.06	4.67 ± 0.97	0.36 ± 0.07
**13f**	0.22 ± 0.04	5.66 ± 1.07	0.40 ± 0.08
**14a**	0.30 ± 0.13	4.16 ± 1.82	14.37 ± 6.28
**14b**	0.25 ± 0.05	5.11 ± 1.08	2.26 ± 0.48
**14c**	0.12 ± 0.03	10.83 ± 2.33	2.52 ± 0.54
**14d**	0.07 ± 0.02	17.94 ± 4.10	2.90 ± 0.66
**14e**	0.05 ± 0.01	23.26 ± 5.17	2.54 ± 0.56
**14f**	0.05 ± 0.01	27.30 ± 6.53	2.46 ± 0.59
ASF	1.26 ± 0.25	1.00 ± 0.20	0.11 ± 0.02

Table S4. Values of K_D in SPR measurements with neo-glycoproteins and immobilized galectin-3 Apparent K_D values determined by SPR are compared for all in the present and recent study designed neo-glycoproteins. Neo-glycoproteins and ASF were flowed over the surfaced immobilized with galectin-3. Values were calculated by fitting association and dissociation using Scrubber2. (n.d. – not detectable)

Ligand	Attached glycans	Apparent K_D [nM]	Ligand	Attached glycans	Apparent K_D [nM]
**13a**	0.5	n.d.	**15a**	1.6	103 ± 3
**13b**	3.3	9.40 ± 0.10	**15b**	7.5	9.80 ± 0.10
**13c**	6.4	4.84 ± 0.05	**15c**	14.4	4.77 ± 0.07
**13d**	9.4	3.12 ± 0.03	**15d**	17.8	3.18 ± 0.06
**13e**	13.0	2.64 ± 0.03	**15e**	24.2	2.35 ± 0.05
**13f**	14.2	2.57 ± 0.02	**15f**	29.0	1.72 ± 0.04
**14a**	0.3	22 ± 1	**16a**	1.7	n.d.
**14b**	2.3	4.17 ± 0.05	**16b**	7.5	9.50 ± 0.10
**14c**	4.3	2.74 ± 0.03	**16c**	14.1	2.11 ± 0.02
**14d**	6.2	1.95 ± 0.02	**16d**	18.0	2.86 ± 0.02
**14e**	9.2	2.01 ± 0.02	**16e**	24.4	1.50 ± 0.20
**14f**	11.1	1.90 ± 0.20	**16f**	27.5	1.28 ± 0.02
			ASF		75 ± 2

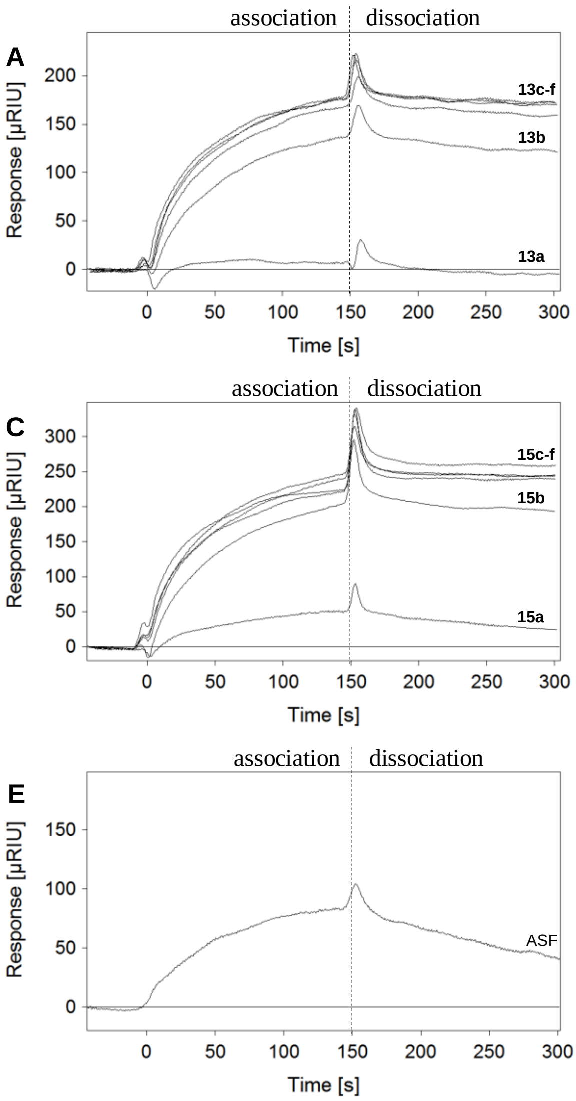

Figure S2. SPR sensorgrams of neo-glycoproteins bound by immobilized galectin-3
Neo-glycoproteins carrying (**A**) 6-biotin LacNAc-LacNAc (**13a-f**), (**B**) 6-biotin LacDiNAc-LacNAc (**14a-f**), (**C**) LacNAc-LacNAc (**15a-f**) and (**D**) LacDiNAc-LacNAc (**16a-f**) as well as ASF (**E**) were applied in flow on the surface immobilized with galectin-3. Responses of different ligands at a concentration of 0.2 µM were plotted against the time. With increasing glycan number per BSA steeper slopes are observed.
